# Annotations

**Published:** 1903-04-18

**Authors:** 


### Annotations.

### The Public Schoolboy's Body.

Perhaps there is no question except religion on which persons apparently equally well qualified to express an opinion may hold such opposite notions as education. This is shown in the *Westminster Review* for March, where Mr. J. H. Vines waxes eloquent over the improvement in the physique of the boys at our public schools, while Mr. P. S. Burrell, in the same journal mourns the evil done by too much education, physical as well as mental. To take the pleasanter point of view first—Mr. Vines brings forward statistics to prove that in both height and weight the schoolboy of to-day is superior to the boy of thirty years ago. He draws his examples from two English schools, Marlborough and Rugby. The Marlborough statistics are the most complete, as they show the average of the whole school, whereas at Rugby the measurements of only the gymnasium boys are taken. The Marlborough measurements compared are those taken in 1874 and 1901, and at every age there is shown an increase both in height and weight. A boy of thirteen of to-day weighs on an average five and a half pounds more than did his predecessor of 1874, and he is two inches taller. The eighteen-year old boy of the present time weighs four and a half pounds more, and is nine-tenths of an inch taller than the boy of the past generation. Indeed, the difference is probably greater than the figures seem to show, for the earlier measurements were taken with shoes on and the latter ones without them. At Rugby the compared measurements are those of 1879 and 1901. These show that the boy who was thirteen in 1901 is two and a half inches taller and rather more than six pounds heavier than the boy of that age was in 1879. At seventeen there is an increase in height of nine-tenths of an inch, but a diminution in weight of a pound. All the measurements bear out the contention that the young Briton is a stronger and healthier human being than his father was.

### The Board School Boy's Mind.

Mr. Burrell on the other hand complains that we have now "too much education." After thirty years of compulsory education there is less contentment, and not much more virtue than before the days of Board School, and we are threatened more than ever by foreign competition. As an adaptation of means to an end, the maintenance of our nation in the premier position in the world, education seems confessedly to have failed. Mr. Burrell points out three errors into which educationists fell. First, children were treated as knowledge boxes, to be stuffed as full as they would hold, and then made repeat what they had been taught with as much understanding as a phonograph. Secondly, town and country children were taught exactly the same things, and these things being more or less of a "towny" kind—or at least of no use in rustic life—education of this sort encouraged the rural exodus, of which we see the results in a depopulated country and overcrowded towns where there is not work for all. Thirdly, originality, enterprise, initiative, and intellectual independence were stifled. The justice of these strictures must be admitted. As for secondary education, the error in it is the attempt to teach "a maximum of subjects in a minimum of time," in order to pass examinations and gain certificates. What Mr. Burrell pleads for is "a little wholesome neglect" for children, a little time in which they can do as they like. School games have a discipline of their own, and teach the virtues of united action, and the subordination of the aims of each to the success of all. But at the same time there should be time for each child to carry out his own particular hobby, make his own blunders, and find out his roads to success Yet, when all is said and done, has our educational system been so great a failure? All over the empire we find men educated in the old country doing good work in a new one. Men from the university and men from the Board School alike are showing that neither system has quenched their energy and their initiative. And if we are lagging behind in the commercial race, it does not follow that Mr. Burrell is right in attributing our slowness to "too much education." The nations which are beating us in the race are those whose sons are better educated than ours—the German and the American.

### Oyster Beds and Sewage Disposal.

Oyster beds, it is contended, should be removed by those interested in the trade in these molluscs to some other place than the estuary of a river that carried down sewage to the sea. This contention naturally provoked considerable discussion at a recent meeting of the Sanitary Institute. Mr. Roesling, of Leicester, protested that to compel the removal of oyster-beds in this way was to make the injured party do the work which should fall on the one who wrought the injury, while the Chairman, Colonel Harding, who is a member of the Commission, declared that Dr. Reid had entirely misconceived the document against which he was arguing. Colonel Harding pointed out that the recommendations regarding the getting rid of bacteria were not recommendations from the Commission, but the advice of experts given to the Commission, who by publishing their opinions did not in any way bind themselves to adopt them. But as Dr. Reid pointed out, the fact that the second report of the Commission was entirely taken up with this expert evidence, very naturally made those who read it think that it expressed the opinion of the Commissioners themselves. In this contention Dr. Reid was supported by Sir Henry Robinson and Dr. Scott Moncrieff, who also agreed with the lecturer, as did Mr. Bostock Hill, that the long delay in the publication of the Commission's finding was causing great inconvenience to sanitary authorities, who found it impossible to ask local bodies to incur any expense for alterations in their methods of sewage disposal, for fear that after a considerable sum of money had been spent, the report of the Commission would result in some other scheme being made compulsory, which would mean that all the money previously expended would be lost. All those who took part in the discussion went on the assumption that sewage must ultimately find its way into our rivers, except Mr. Roesling, who not only spoke enthusiastically of the results of the land treatment of sewage as shown abroad, but claimed that in Leicester they had proved that even a clay soil, though it was generally looked upon as practically impermeable, could be used successfully for that purpose.

